# Red blood cell distribution width as a novel prognostic marker after myocardial revascularization or cardiac valve surgery

**DOI:** 10.1038/s41598-021-87075-4

**Published:** 2021-04-12

**Authors:** Davide Lazzeroni, Luca Moderato, P. L. Marazzi, Carmen Pellegrino, Elisa Musiari, Paolo Castiglioni, Umberto Camaiora, Matteo Bini, Simone Geroldi, Lorenzo Brambilla, Valerio Brambilla, Paolo Coruzzi

**Affiliations:** 1 IRCCS Fondazione Don Carlo Gnocchi, Milan, Italy; 2 Guglielmo da Saliceto Hospital, Piacenza, Italy; 3 Fondazione Don Carlo Gnocchi, Parma, Italy; 4 Department of Medicine and Surgery, University of Parma, Parma, Italy; 5 Piazzale dei Servi, n°3, 43121 Parma, Italy

**Keywords:** Cardiology, Outcomes research

## Abstract

The red blood cell distribution width (RDW) measures the variability in the size of circulating erythrocytes. Previous studies suggested a powerful correlation between RDW obtained from a standard complete blood count and cardiovascular diseases in both primary and secondary cardiovascular prevention. The current study aimed to evaluate the prognostic role of RDW in patients undergoing cardiac rehabilitation after myocardial revascularization and/or cardiac valve surgery. The study included 1.031 patients with available RDW levels, prospectively followed for a mean of 4.5 ± 3.5 years. The mean age was 68 ± 12 years, the mean RDW was 14.7 ± 1.8%; 492 patients (48%) underwent cardiac rehabilitation after myocardial revascularization, 371 (36%) after cardiac valve surgery, 102 (10%) after valve-plus-coronary artery by-pass graft surgery, 66 (6%) for other indications. Kaplan–Meier analysis and Cox hazard analysis were used to associate RDW with mortality. Kaplan–Meier analysis demonstrated worse survival curves free from overall (log-rank p < 0.0001) and cardiovascular (log-rank p < 0.0001) mortality in the highest RDW tertile. Cox analysis showed RDW levels correlated significantly with the probability of overall (HR 1.26; 95% CI 1.19–1.32; p < 0.001) and cardiovascular (HR 1.31; 95% CI 1.23–1.40; p < 0.001) mortality. After multiple adjustments for cardiovascular risk factors, hemoglobin, hematocrit, C-reactive protein, microalbuminuria, atrial fibrillation, glomerular filtration rate,left ventricular ejection fraction and number of exercise training sessions attended, the increased risk of overall (HR 1.10; 95% CI 1.01–1.27; p = 0.039) and cardiovascular (HR 1.13; 95% CI 1.01–1.34; p = 0.036)mortality with increasing RDW values remained significant. The RDW represents an independent predictor of overall and cardiovascular mortality in secondary cardiovascular prevention patients undergoing cardiac rehabilitation.

## Introduction

Red blood cell distribution width (RDW), obtained from a standard complete blood count, represents a measure of the variability in size of circulating erythrocytes and is indicated as the coefficient of variation of the erythrocyte size^1^. RDW is the ratio between standard deviation (SD) and mean of the mean corpuscular volume; the ratio is multiplied by 100 to yield a percentage value^1^. The normal reference range of RDW is 11–15%^2^ and values > 15% indicate the presence of anisocytosis. Recently, high RDW percentage values have been associated with the progression and the severity of CV diseases^3^. Several possible pathogenetic mechanisms have been proposed including microvascular disorder^4^, anemia^5,6^, inflammatory cytokines^7^, oxidative stress^8^, free cholesterol^9^, thrombosis^10,11^, nutritional deficiency^12^, renal dysfunction^13^, and hyper-adrenergic tone^14^. Moreover, correlations were also found between RDW and increased risk of hypertension^15,16^, atrial fibrillation^12^, myocardial infarction^8^, heart failure^17^, stroke^18^ and mortality^19,20^*.* On the other hand, no studies have so far evaluated whether RDW may play a role in the long-term outcome of patients undergoing cardiac rehabilitation programme after cardiac surgery; therefore, the present work aimed to elucidate this issue.

## Methods

We considered all the consecutive patients admitted to our rehabilitation department between January 2007 and June 2015 to undergo cardiac rehabilitation (CR) after cardiac surgery. Clinical indication for CR were the following: myocardial revascularization by coronary artery bypass graft (CABG), valve surgery and valve and CABG surgery. For the present study we included the 1031 patients with available RDW levels, after excluding patients who underent CR after percutaneous coronary intervention, percutaneous valve replacement, heart failure hospitalization, patients who have not completed the CR programme due to complications that required intensive care unit as well as those without available RDW levels. All the enrolled patients provided written informed consent. The ethics committee on human research of the IRCCS Fondazione Don C. Gnocchi, Milan (Italy) approved the study that was carried out in accordance with the Declaration of Helsinki.

All the patients completed a standard in-hospital CR program, lasting approximately 2 weeks, consisting of supervised exercise sessions (120 min per day), lifestyle and risk factor management, counseling, and medical therapy optimization. Anamnestic data and demographics, clinical and laboratory variables including RDW levels, electrocardiographic and echocardiographic measurements, coronary angiography data, physical activity parameters and pharmacological therapy adherence were collected for each patient at the discharge from the CR program. Coronary artery disease was assessed by coronary angiography during hospitalization before cardiac surgery. At discharge to the CR program after surgery, in all the patients a standard 12-lead ECG (Mortara Instrument-Portrait) and a standard adult transthoracic echocardiography (EsaoteMyLab 60) was performed according to the American Society of Echocardiography (ASE) recommendations^21^.

Outcomes during follow-up were collected periodically (every 2 years) by a medical doctor through telephone-administered questionnaires. Whenever we received information about any event after the telephone call, we verified the nature of the event on the hospital clinical records and/or contacted the general practitioner. Endpoints were overall and cardiovascular (CV) mortality. CV death was identified only in the presence of clear data demonstrating sudden cardiac death or death resulting from acute myocardial infarction, or heart failure or stroke. In the absence of clear data on the modality of death, the event was not classified as cardiovascular death.

### Statistical analysis

We represented continuous variables by the mean (M) and standard deviation (SD) over the group and tested the differences among groups by the analysis of variance (ANOVA), applying the least-significant difference test as post-hoc analysis. We expressed categorical variables in percentage (%) and tested the differences among groups by the Pearson chi-square test. We considered RDW, the predictor variable, both as a continuous and categorical quantity. In the latter case we expressed it as the tertile of its distribution and evaluated the hazard ratio (HR) separately for overall mortality and cardiovascular mortality by the Cox proportional hazard analysis.

We considered a model of covariates in the Cox proportional hazard analysis applied with RDW as a predictor of events to take into account the differences among the RDW tertiles in the patients’ characteristics and known prognostic markers in secondary cardiovascular prevention. The model included age, gender, weight, height, history of arterial hypertension, hyperlipidemia, diabetes, smoking, hemoglobin, hematocrit, C-reactive protein (CRP), microalbuminuria, atrial fibrillation, glomerular filtration rate (GRF) and left ventricular ejection fraction (LV-EF) as covariates. Event-free survival time between the admission and the event was measured and Kaplan–Meier analysis created event-free survival curves among RDW tertiles. Statistical significance was set at p < 0.05. All statistics were performed with SPSS version 24 (IBM Corporation, Armonk, NY, USA).

## Results

### Demographic and clinical characteristics

Among the included 1.031 patients, 724 patients (71%) reported arterial hypertension and 251 (25%) type 2 diabetes; 492 patients (48%) underwent myocardial revascularization by coronary artery bypass graft (CABG), 371 (36%) valve surgery, 102 (10%) valve and CABG surgery, and 66 patients (6%) underwent surgery for other clinical indications (e.g. tricuspid valve disease, aortic aneurysm, atrial masses). Among myocardial revascularization, 59% underwent cardiac surgery after acute coronary syndrome (ACS) and 41% after stable angina or silent ischemia. Among the patients who received valve surgery, 31% underwent surgery for mitral disease, 59% for aortic disease and 10% for both mitral and aortic disease. Number of exercise training sessions attended were 25 ± 5 (two separate sessions: one in the morning and one in the afternoon).

The RDW mean value ± standard deviation was 14.7 ± 1.8% in the whole group of patients. The ANOVA test revealed significant differences among subgroups (p < 0.001) being the RDW value in the CABG subgroup (14.5 ± 1.8%) significantly lower than in the valve surgery subgroup (14.8 ± 1.9%, p = 0.012), in the combined valve-and-CABG surgery subgroup (15.2 ± 1.8%, p = 0.002) as well as in the subgroup operated for other clinical indications (15.2 ± 1.9%, p = 0.002).

The RDW tertiles ranged between 11.2 and 13.8%, between 13.9 and 14.9%, and between 15.0 and 28.6%. The mean follow-up was 4.5 ± 3.5 years, during which there were 146 deaths (14.2%) and 66 cardiovascular deaths (6.9%). Atrial fibrillation at discharge was found in 142 patients (14%).

Patients characteristics by RDW tertiles are reported in Table 1. Patients with elevated RDW (highest tertile) showed a higher prevalence of female gender, arterial hypertension, and smoking habit, older age and lower height and weight. No differences between groups were found in the rate of cardiovascular drugs except for a lower rate of antiplatelet drugs as well as a higher rate of anticoagulants in the highest tertile.

**Table 1:** Patients’ characteristics by tertile of red blood cell distribution width (RDW).

RDW tertile	Lowest (N = 344)	Intermediate (N = 343)	Highest (N = 344)	p value
Tertile range (%)	11.2 – 13.8	13.9 – 14.9	15 – 28.6	
**Personal and anthropometric data**				
Age (years)	63 (12)	69 (10)^§^	71 (10)*	< 0.001
Female sex (%)	18	28^§^	41*	< 0.001
Height (m)	1.72 (0.08)	1.69 (0.08)^§^	1.67 (0.8)*	< 0.001
Weight (Kg)	77 (14)	74 (13)^§^	71 (14)*	< 0.001
**Cardiovascular risk factors**				
Arterial hypertension (%)	32	72^§^	79*	< 0.001
Diabetes (%)	21	25	28	0.052
Smoking habit (%)	26	15^§^	10*	< 0.001
Dyslipidemia (%)	57	54	50	0.132
**Target organ damage**				
Atrial fibrillation at discharge (%)	5.6	13	24	< 0.001
Left ventricle ejection fraction (%)	54 (9)	53 (10)	51 (12)*	< 0.001
Left ventricle mass index (g/m^2^)	110 (41)	117 (37)	117 (38)	0.141
Glomerular filtration rate (mL/min)	93 (34)	76 (26)^§^	69 (30)*	< 0.001
Microalbuminuria (mg/L)	4.4 (3.5)	5.3 (4.7)^§^	5.3 (5.1)*	0.005
**Laboratory data**				
Hemoglobin (g/dL)	11 (1.4)	10 (1.2)^§^	10 (1.2)*	< 0.001
Hematocrit (%)	34 (4)	33 (15)	33 (17)	0.639
C-reactive protein (mg/L)	3.9 (2.3)	4.4 (3.1)	3.9 (2.7)	0.189
Total cholesterol (mg/dL)	124 (32)	125 (36)	132 (41)	0.015
HDL cholesterol (mg/dL)	30 (7)	32 (8)	34 (10)*	< 0.001
LDL cholesterol (mg/dL)	76 (29)	78 (32)	70 (26)	0.538
Triglyceride (mg/dL)	108 (46)	106 (44)	112 (47)	0.297
**Medical therapy**				
ACE-i (%)	48	46	40	0.074
Angiotensin receptor blockers (%)	11	9	10	0.728
Beta blockers (%)	85	83	80	0.138
Calcium channel blockers (%)	6	6	8	0.429
Statins (%)	70	68	65	0.479
Anticoagulants (%)	30	36	45*	< 0.001
Antiplatelets (%)	75	74	67*	0.041

Continuous variables as mean (standard deviation), categorical variables as percentage (%). *HDL* high-density lipoprotein, *LDL* low-density lipoprotein, *ACE-I* angiotensin-converting-enzyme inhibitors; p values after ANOVA for continuous variables, after chi-square test for categorical variables. *Significant differences between the lowest and the highest tertile, ^§^differences between the lowest and the intermediate tertile at p < 0.05.

Patients in the third RDW tertile also showed higher levels of microalbuminuria, total and HDL cholesterol, higher rate of atrial fibrillation at dicharge, as well as lower values of glomerular filtration rate, hemoglobin, and lower left ventricular ejection fraction.

### RDW and mortality

**Figure 1:**
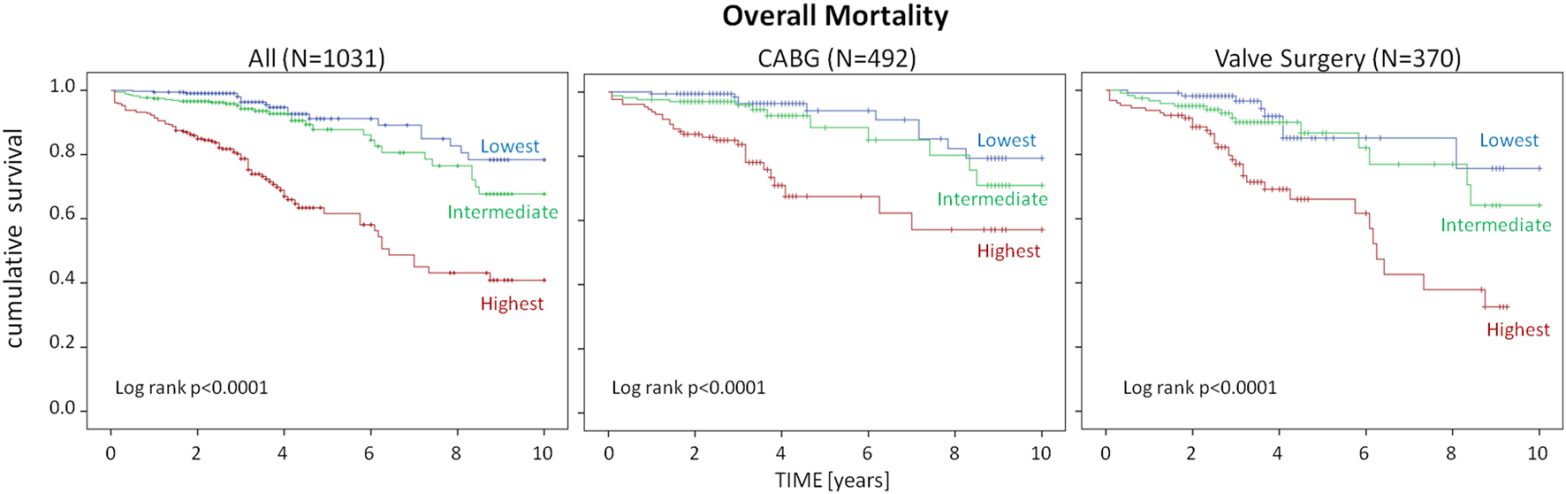
Kaplan–Meier survival curves for overall mortality by tertiles of red blood cell distribution width for the whole patients' population (left panel) and for the subgroups of patients who underwent coronary artery by-pass alone (CABG, central panel) or valve surgery alone (right panel).

**Figure 2:**
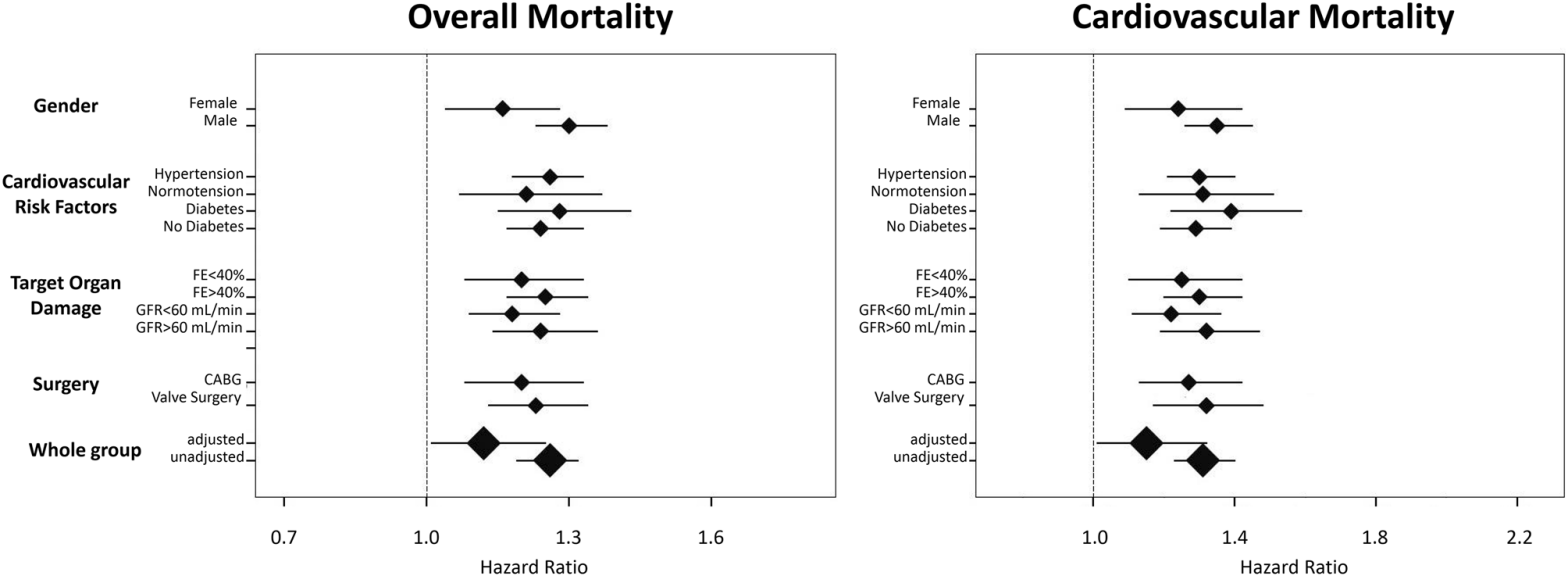
Hazard ratio from Cox proportional analysis describing the risk of death and cardiovascular death in the whole (unadjusted and adjusted) group of patients and in specific sub-groups for each percentage increase of red blood cell distribution width*.*

A significantly higher rate of overall (5% vs 9% vs 28%; p < 0.001) and cardiovascular mortality (1% vs 5% vs 16%; p < 0.001) were found in the highest tertile of RDW compared to the first and second tertiles. Kaplan–Meier analysis demonstrated worse survival curves free from overall (log-rank p < 0.0001) and cardiovascular (log-rank p < 0.0001) mortality in the highest RDW tertile in the overall population, as well as in patients who received CABG surgery alone (log-rank p < 0.0001) and valve surgery alone (log-rank p < 0.0001) (Fig. 1). Considered also as a continuous variable RDW correlated directly and significantly with the probability of overall mortality (HR 1.26; 95% CI 1.19–1.32; p < 0.001), corresponding to a 26% increase of the relative risk of death per unitary of RDW increase. Similarly, RDW correlated directly and significantly with the probability of cardiovascular mortality (HR 1.31; 95% CI 1.23–1.40; p < 0.001), corresponding to a 31% increase of the relative risk of CV death per unitary of RDW increase. Interestingly, after dividing the patients' population according to gender, arterial hypertension, diabetes, glomerular filtration rate (cut-off 60 ml/min), left ventricular ejection fraction (cut-off 40%), and type of intervention (myocardial revascularization in both acute or chronic coronary syndrome and valve surgery), overall and cardiovascular mortality risk rate were significantly higher with the progressive increase of RDW levels in all sub-groups (Fig. 2). Moreover, a significantly increased risk of overall mortality (HR 1.10; 95% CI 1.01–1.27; p = 0.039) and cardiovascular (HR = 1.13; 95% CI 1.01–1.34; p = 0.036) was found even after multiple adjustments for age, gender, weight, height, history of arterial hypertension, dyslipidemia, diabetes, smoking, heamoglobin, hematocrit, C-reactive protein, atrial fibrillation, microalbuminuria, GFR, LV-EF and number of exercise training sessions attended.

## Discussion

Our study is among the few investigating the predictive value of RDW after a period of cardiac rehabilitation following cardiac surgery in both ischemic and valve disease patients. Our results demonstrate a role of RDW percentage values in predicting overall and cardiovascular mortality independent from gender, arterial hypertension, diabetes, GFR, LVEF and type of intervention.

Although RDW was historically used as a diagnostic marker of hematological disorders (such as chronic anemia due to vitamins deficiency and liver or kidney failure), the role of red blood cells biology in the pathogenesis of non-hematological disorders has been recently evaluated and a correlation between RDW and increased risk of hypertension^15,16^, atrial fibrillation^12^, myocardial infarction^8^, heart failure^17^, stroke^18^ and mortality^19,20^ have been reported.

Skjelbakken et al., investigating the association between RDW and the risk of the first-ever event of myocardial infarction in 25.612 participants, found that, independently of anemia and cardiovascular risk factors, RDW was associated with incident myocardial infarction in a general population^8^. A post-hoc analysis from the Cholesterol And Recurrent Events (CARE) on 4.111 participants in the Tromsø study with available RDW data (follow-up of 59.7 months) reported a graded, independent relationship between higher levels of RDW and the risk of death and cardiovascular events in people with prior myocardial infarction^22^.

Furthermore, Warwick et al.^23^ found that pre-operative RDW represents a significant determinant of in-hospital mortality and long-term survival in 8.615 patients undergoing isolated CABG (median follow up of 5.8 years) and our data are in line with the above-cited results, even when RDW was examined in the post-surgical period during the cardiac rehabilitation programme.

A retrospective, observational work investigating 214 consecutive patients with unstable angina pectoris who underwent successful percutaneous coronary interventions, also showed a strong association between baseline RDW and in-stent restenosis^24^ as well as a study aimed to identify potential laboratory markers in 2.679 symptomatic chronic heart failure patients from the North American CHARM (Candesartan in Heart Failure: Assessment of Reduction in Mortality and Morbidity) program, demonstrated that higher RDW is associated with morbidity and mortality^17^. In particular, a higher RDW did result among the most powerful predictors, age and cardiomegaly only showing a better independent association with outcome^17^.

The prognostic value of elevated RDW was also confirmed in patients undergoing valve replacement or repair surgery as demonstrated by a higher risk of transient ischaemic attack, early peri-operative stroke, and death^25^. Moreover, the evaluation of the short- and long-term prognostic value of RDW in a large cohort of transcatheter aortic valve implantation patients revealed that elevated RDW represents a strong independent marker and predictor of short- and long-term mortality^26^. Experimental and human studies have proposed microvascular dysfunction^4^, anemia^5,6^, inflammatory cytokines^7^, oxidative stress^8^, free cholesterol^9^, thrombosis^10,11^, nutritional deficiency^12^, renal failure^13^, and autonomic dysfunction^14^ as possible pathophysiological mechanisms linking RDW with CV diseases.

**Figure 3:**
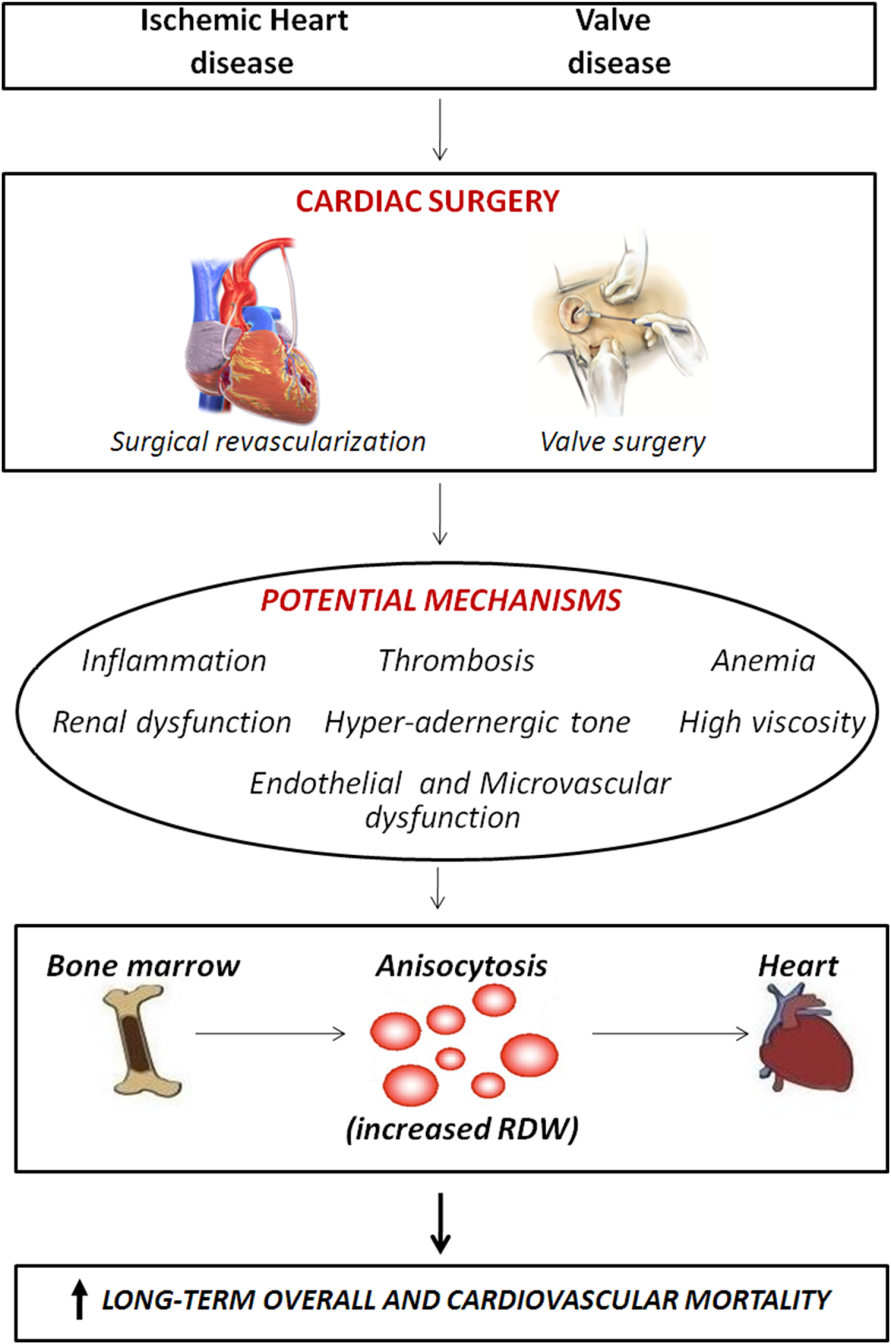
Possible patho-physiological mechanisms linking anysocitosis to long-term mortality after cardiac surgery.

Interestingly, beta-blockers administration^27^, as well as 3-week exercise training^28^, both powerful modulators of autonomic activity and functional capacity during cardiac rehabilitation^29^, did result associated with a significant reduction of RDW levels, thereby supporting an autonomic pathogenetic hypothesis. More recently some scheming scenarios have been opened in the field of CV medicine where RDW seems to behave not only as a marker of pathological processes but also as a possible active actor. In fact, anisocytosis has been shown to be positively associated with decreased red blood cell deformability, thus contributing to impair microvascular function by decreasing blood viscosity^30^. Furthermore, the accumulation of free cholesterol driving from the red blood cell membrane has been reported as increased in subjects with high, anisocytosis levels^9^ promoting the expansion of the lipid core^31^. Moreover,

during the atherosclerosis process, the iron released as a consequence of erythrophagocytosis or mediated by erythrocyte injury, may also amplify the formation of foam cells, thus promoting the growth and the instability of the atherosclerotic plaque^32,33^. Finally, nitric oxide released by cell-free hemoglobin upon injury of erythrocytes within the necrotic core of the atherosclerotic plaque may also contribute to inhibiting endothelium-dependent vasodilation^34^. Even though our data do not allow to provide a pathophysiological explanation, we may speculate that some of the above-mentioned mechanisms, especially hyper-adrenergic tone, inflammation, and endothelial dysfunction, may persist throughout rehabilitation period following cardiac surgery. In particular, in operated cardiac patients (e.g., for CABG or valve repair/replacement), cardiac surgery could activate a series of potentially negative mechanisms (inflammation, hyper adrenergic tone, etc.) contributing to induce bone marrow to produce red blood cells with a wide width distribution (anysocitosis) and to increase long-term mortality (Fig. 3).

Some methodological limitations should be highlighted: (a) pre-existing hematological diseases and pre-operative RDW measures are lacking, (b) RDW was measured only at discharge from cardiac rehabilitation programme, (c) data were adjusted considering hematocrit and hemoglobin values while neutrophil/lymphocyte ratio and platelets counts were not collected, (d) long-term RDW measurement is lacking, (e) peri- and postoperative transfusion data are not available, (f) the exact etiologies of non-cardiovascular deaths are lacking, (f) lack of a control group of subjects that did not attend CR to assess its relative contribution on RDW and outcomes, (g) lack of functional improvement during CR (between admission and discharge) to assess the impact of exercise based programme on RDW and outcomes, (h) a loss to follow-up bias cannot be excluded since 24% of patients were lost at the last follow-up session, (i) finally, an intrinsic risk of over-adjustment cannot be excluded in the presence of a broad multivariate analysis. In conclusion, our data demonstrate that RDW represents an independent predictor of overall and cardiovascular mortality in patients undergoing cardiac rehabilitation after myocardial revascularization or cardiac valve surgery (in secondary cardiovascular prevention patients undergoing cardiac rehabilitation).
